# Influence of N6-Benzyladenine and Sucrose on *In Vitro* Direct Regeneration and Microrhizome Induction of *Kaempferia parviflora* Wall. Ex Baker, An Important Ethnomedicinal Herb of Asia

**DOI:** 10.21315/tlsr2020.31.1.8

**Published:** 2020-04-07

**Authors:** Catherine Labrooy, Thohirah Lee Abdullah, Johnson Stanslas

**Affiliations:** 1 Department of Crop Science, Faculty of Agriculture, Universiti Putra Malaysia, 43400 Serdang, Selangor, Malaysia; 2 Department of Medicine, Faculty of Medicine and Health Sciences, Universiti Putra Malaysia, 43400 Serdang, Selangor, Malaysia

**Keywords:** Acclimatisation, BA, Multiple shoot, Single factor, Sucrose, Aklimatisasi, BA, Pucuk berganda, Faktor tunggal, Sukrosa

## Abstract

*Kaempferia parviflora* is an ethnomedicinally important plant*.* Conventional propagation of *K. parviflora* is hindered by slow growth rate, long dormancy periods and dual use of rhizomes for seeds as well as marketable produce*.* In our study, we developed a promising dual-phase micropropagation protocol to increase number of plantlets, survivability, biomass and quality plantlets for mass production. Multiple shoot regeneration was found most successful on Murashige and Skoog (MS) media supplemented with 35.52 μM N6-benzyladenine (BA) in terms of highest number of shoots (22.4 ± 1.84), leaves (29.27 ± 1.30), and roots (17.8 ± 1.72) per explant. High survivability was observed with an acclimatisation percentage of 100% in sterile perlite medium. This method was shown to be preferable compared to conventional propagation in terms of propagation time and number of plantlets. Regenerated *in vitro* plantlets were then successfully induced to form microrhizomes in MS media with an optimal concentration of 6% (w/v) sucrose. Increase in microrhizome biomass (35.7 ± 2.59 g per flask), number of microrhizomes (5.2 ± 0.78), shoots (8.5 ± 1.58) and roots (8.5 ± 1.58) were observed for this treatment. This investigation successfully highlights the manipulation of single factors in short time frame to produce a simple and efficient alternative propagation method for *K. parviflora*.

## Abstract (ms)

*Kaempferia parviflora* adalah tumbuhan yang penting dari segi “ethnomedical”. Penghasilan *K. parviflora* secara konvensional dihindar oleh kadar pertumbuhan yang lambat, tempoh dorman yang panjang dan penggunaan rimpang sebagai benih dan hasil yang boleh dipasarkan. Dalam kajian ini, protokol tisu kultur secara dual fasa dapat meningkatkan bilangan tumbuhan, aklimatisasi tinggi, serta peningkatan biomas dan penghasilan tumbuhan berkualiti untuk pengeluaran besar-besaran. Pucuk berganda dihasilkan dengan banyak di media Murashige dan Skoog (MS) ditambah dengan 35.52 μM N6-benzyladenine (BA). Ini diperhati dari segi jumlah pucuk tertinggi (22.4 ± 1.84), bilangan daun (29.27 ± 1.30), dan bilangan akar (17.8 ± 1.72). Ketahanan hidup yang tinggi diperhatikan dengan peratusan aklimatisasi 100% dalam medium perlit steril. Kaedah ini ditunjukkan lebih baik berbanding dengan penanaman konvensional dari segi masa penanaman dan bilangan tanaman. Tumbuh-tumbuhan *in vitro* telah berjaya diinduksi untuk membentuk rimpang mikro dalam media MS dengan kepekatan 6% (w/v) sukrosa. Peningkatan berat rimpang mikro (35.7 ± 2.59 g setiap lobis), bilangan rimpang mikro (5.2 ± 0.78), bilangan pucuk (8.5 ± 1.58) dan bilangan akar (8.5 ± 1.58) yang paling banyak diperhatikan untuk rawatan ini. Penyiasatan ini berjaya menonjolkan manipulasi faktor tunggal dalam jangka masa pendek untuk menghasilkan kaedah penyebaran alternatif yang mudah dan cekap untuk *K. parviflora.*

**Highlights**

- Multiple shoot regeneration was found most successful on Murashige and Skoog (MS) media supplemented with 35.52 μM N6-benzyladenine.
- High survivability was observed with an acclimatisation percentage of 100%.
- Microrhizomes were successfully induced in MS media with an optimal concentration of 6% (w/v) sucrose.

## INTRODUCTION

**Figure 1:**
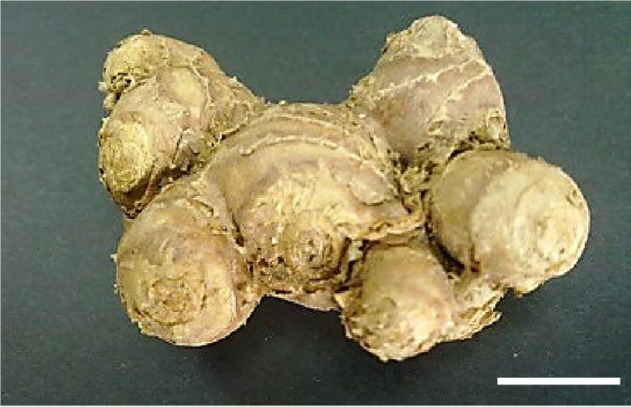

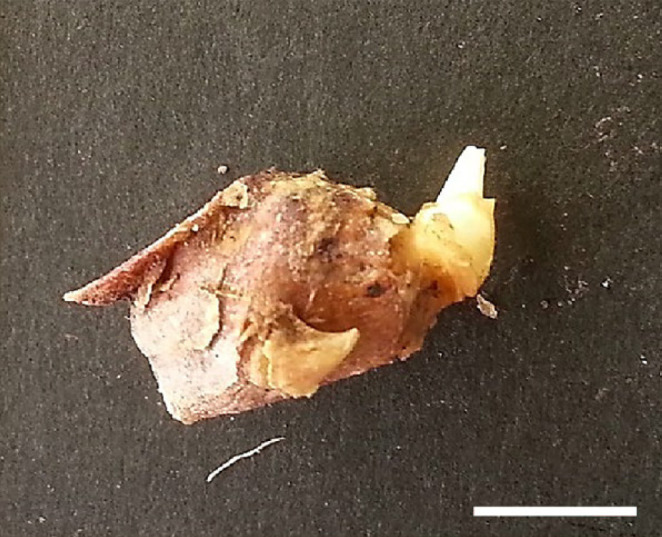

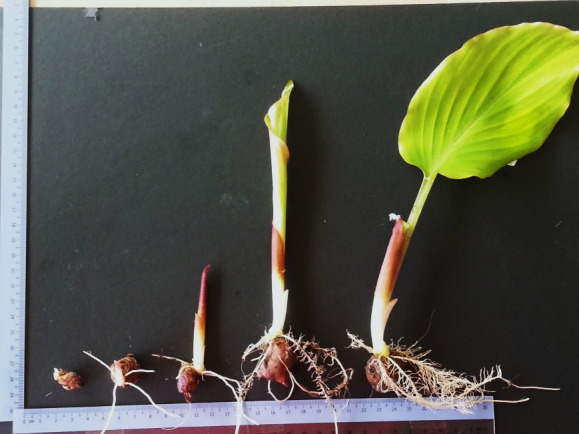

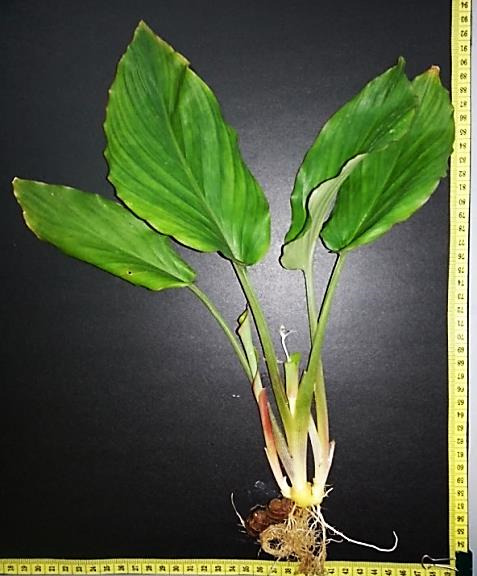

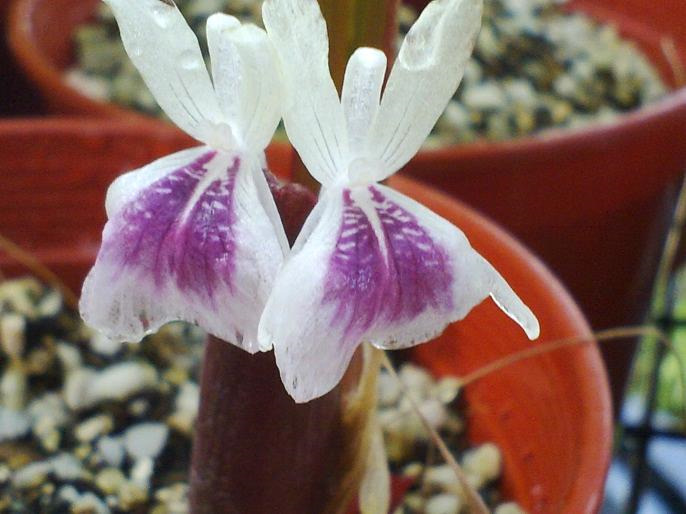

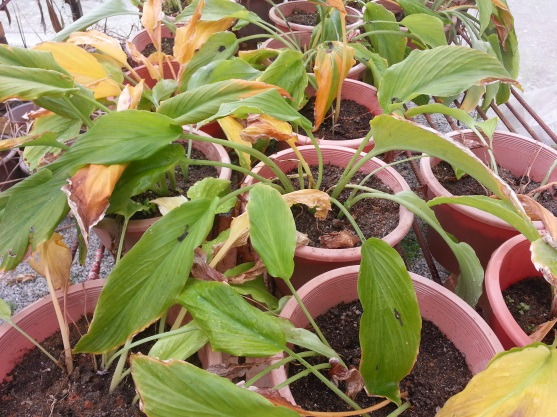
*K. parviflora* growth cycle via conventional propagation: (a) Dormant rhizome; (b) Rhizome with visible bud sprout; (c) Vegetative growth stage of rhizome bud sprouting up to one unfurled leaf stage. This takes approximately ± 120 days. (d) This is a mature adult plant with several unfurled leaves about to start flowering stage at ± 155 days. (e) The true flower of *K. parviflora*. The flowering stage lasts about ± 45 days. (f) At the end of the growth stage ± 240 days, the plant undergoes senescence and the above ground parts dry up as the rhizomes enter dormancy period. (Scale —— represents 1 cm).

The black galingale, *cekur hitam* in Malay and *Krachaidam* in Thai are common names of *Kaempferia parviflora* Wall. ex Baker. A perennial herb with deep purple to black rhizomes that distinguishes it from other members of the Zingiberaceae family and traditionally used for treating gastrointestinal disorders, fungal infections, allergies, and to increase vitality (Yenjai *et al.* 2004; Rujjanawate *et al.* 2005; Tewtrakul *et al.* 2008; Trisomboon 2009). Recent ethnomedicinal research focused on this plant has increased its popularity and demand. Studies have reported a myriad of bioactivity including anti-allergic (Tewtrakul *et al.* 2008), anti-inflammatory (Sae-wong *et al.* 2009), anti-fungal, anti-plasmodial, anti-mycobacterial (Yenjai *et al.* 2009), anti HIV-1 protease activity (Sookkongwaree *et al.* 2006), vasorelaxation and antispasmodic effects (Wattanapitayakul *et al.* 2008). *K. parviflora*’s health promoting benefits and potential therapeutic functions increases its marketability as herbal products. It is also well known as an aphrodisiac and sometimes referred to as Thai Ginseng. But despite the high demand for the rhizomes of *K. parviflora*, there is a scarcity of its planting materials in Malaysia (Noor Camellia 2012). This is due to sluggish propagation of *K. parviflora* through rhizome splitting along with a long dormancy period, which further restricts the use of *K. parviflora* for large scale cultivation. *K. parviflora* has a growth cycle of 9 months (Fig. 1).

Dormant rhizomes with terminal buds are split and sprouted (Figs. 1a and 1b). The vegetative growth can take up to 3 months and the reproductive stage lasts about 1 to 2 months more (Figs. 1c and 1d). The flowers are inconspicuous and does not set seed (Fig. 1e). Aerial plant parts dry up and dormancy phase sets in (Fig. 1f). The rhizomes are then harvested and marketed while some are kept as planting material for the next planting season. The long dormancy period affects cropping cycles, year-round cultivation and is a major impediment in the commercial cultivation of this plant. It restricts year-round availability of the crop. Under natural conditions in Thailand, *K. parviflora* plants undergo a dormancy phase for five to six months from November to early May during a dry season (Techaprasan *et al.* 2010). A similar dormancy pattern was observed in *K. perviflora* grown conventionally in Malaysia (Fig. 1).

Propagation by rhizome splitting is also limited because only fewer number of propagules can be obtained simultaneously from a single plant. In *Kaempferia galanga* it has been reported that a maximum of 2 to 4 plants can be obtained in a year from a single rhizome (Chirangini *et al*. 2005). Therefore, to overcome obstacles such as year-round availability and increase the amount of high quality planting material, micropropagation or *in vitro* mass multiplication in *K. parviflora* was investigated. *In vitro* studies in *K. parviflora* are limited with only few known reports (Dheeranupattana *et al.* 2003; Mongkolchaipak *et al*. 2006*;* Prathanturarug *et al.* 2007; Alveno 2012; Zuraida *et al.* 2014) whereas report on microrhizome production is minimal. Microrhizome production is a technique of inducing rhizomes under *in vitro* conditions. Microrhizomes are beneficial because they can help prevent misidentification, minimise acclimatisation time, serve as direct planting materials and can also be used as a source for secondary metabolites. Microrhizomes are also easy to store, less vulnerable to transport and international shipping and are suitable for germplasm conservation (Sharma & Singh 1995). This study introduces simple protocols for micropropagation and microzhizome induction of *K. parviflora* by manipulating only a single factor in terms of plant growth regulator (PGR) or carbohydrate levels. This easy and cost-effective production system will benefit producers and improve availability of high quality *K. parviflora* propagules especially in the Malaysian herb economy.

## MATERIALS AND METHODS

### Plant Materials

*K. parviflora ex vitro* rhizomes were obtained from Pusat Herba Universiti Putra Malaysia (UPM), Serdang, Selangor. A voucher specimen was deposited in the herbarium of Institute of Bioscience (IBS) UPM with the following voucher number SK2537/14.

### Pre-sterilisation

Rhizomes were washed thoroughly with running water to remove any soil residue. A soft brush was used to scrub out any remaining dirt and scale on the rhizome surface. After rinsing with distilled water, the rhizomes were soaked in 10% Bentlate solution for one hour with constant agitation. Rhizomes were then sprouted for 14 days on autoclaved perlite to reduce contamination. Rhizomatous buds were isolated once they protruded from the dried rhizome skin and were visibly green. Buds were cut out from the rhizome in 1 cm (approx.) size and used as explants. This procedure was done to minimise the presence of soil borne bacteria and fungus.

### Surface Disinfection

Excised buds were washed in 1 L water with 3 drops of full strength Teepol® (R&M chemicals, Essex, UK) and for 1 min and then rinsed in distilled water thrice. In the laminar flow, explants were soaked in 10% (v/v) Clorox® (5.25% Sodium hypochlo-rite) [Clorox (M) Pte. Ltd., Rawang, Malaysia] with 3 drops Tween 20 (Merck Schuchardt, Hobenburn, Germany) for 5 min then rinsed thrice in sterile water, soaked in 0.1% (v/v) mercuric chloride for 3 min then rinsed 5 times with water. Explants were trimmed from the end and immediately used for regeneration experiment.

### Multiple Shoot Regeneration

Disinfected rhizome buds were inoculated on Murashige and Skoog (MS) (1962) medium supplemented with 3% (w/v) sucrose, 0.5% (w/v) Gelrite™ (Duchefa, Haarlem, The Netherlands), and different concentrations of N6-benzyladenine (BA) (0, 8.88,17.76, 26.64, 35.52, and 44.40 μM). The pH of the media was adjusted to 5.8. The media was distributed into vials (10 mL each) before autoclaving at 121°C at 103 kgm^−1^s^−2^ for 15 min. After inoculation the explants were incubated at 25 ± 2°C, with 16 h photoperiod (30 μmol m^−2^ s^−1^). Cultures were maintained on the same media by subculturing at 3-week intervals for 9 weeks. After the third week subculturing was done in culture vessels (50 mL media). Fifteen explants were used per treatment.

### Rooting

Rooting was spontaneous on MSO (MS devoid of any PGR) media at 3 to 4 weeks after shoot multiplication. Rooted cultures were maintained in the same media by regular subculturing at 3-week intervals for further experiments.

### Microrhizome Induction

Regenerated aseptic shoots (2 ± 0.5 cm long) were used as explants to induce microrhizome formation. The explants were subcultured into 150 mL flasks with 50 mL MS medium supplemented with varying concentration of sucrose [3% (w/v), 6% (w/v) and 9% (w/v)]. The cultures were incubated at 25 ± 2°C, with 16 h photoperiod (30 μmol m^−2^ s^−1^) for 9 weeks with subculturing done at 3-week intervals. Data was collected at the end of 9 weeks of culture.

### Acclimatisation

For the first study, well rooted shoots, around 3 cm long having 3 to 4 leaves were rinsed with sterile water to remove residual MSO (MS devoid of any PGR) medium. The plantlets were then transferred to pots (8 cm height × 10.5 cm diameter) containing autoclaved perlite for primary acclimatisation. The plantlets were sprayed with 0.5% (w/v) Benocide 50WT® (Hextar Chemicals Pte. Ltd., Klang, Malaysia) to reduce microbial contamination. To maintain humidity, the plantlets were covered with transparent plastic sheets and incubated at 27 ± 3°C with 16 h photoperiod (30 μmol m^−2^ s^−1^). Watering was done every alternate day with distilled water. After 2 weeks, the plantlets were transferred to the field and placed in a mist house for secondary acclimatisation. In the second study, shoots with microrhizomes were rinsed with sterile distilled water to remove residual MSO medium. Similar antifungal treatment was given and plantlets with microrhizomes were directly transferred to the mist house.

## STATISTICAL ANALYSIS

Experimentations were replicated thrice comprising 15 samples for each replication following completely randomised design. Data were subjected to analysis of variance (ANOVA) using SAS version 9.4. When treatments were significant, means were separated using Duncan’s Multiple Test Range (DMRT) (Duncan 1955). Statistical significance was considered at *P* ≤ 0.05.

## RESULTS

### Multiple Shoot and Root Regeneration

**Figure 2:**
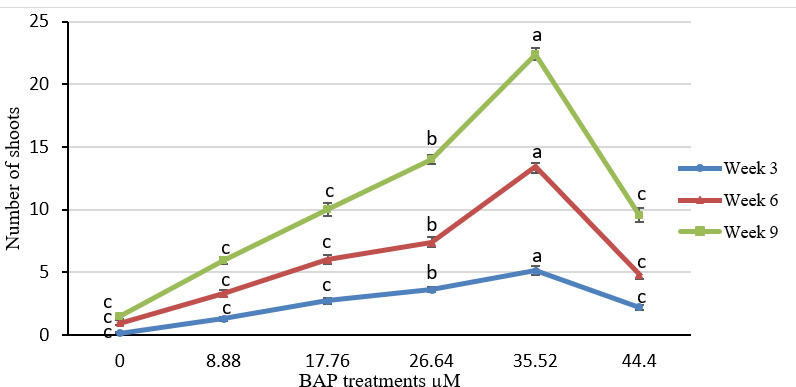
*K. parviflora* growth under different 6-benzyladenine (BAP) concentrations estimated by number of shoots at 3, 6 and 9 weeks of culture. Results represent mean ± standard error of 15 replicates (*P* ≤ 0.05). Error bars represent standard error. Treatment of 35.52 μM BAP showed highest number of shoots (22) after 9 weeks of treatment.

**Figure 3:**
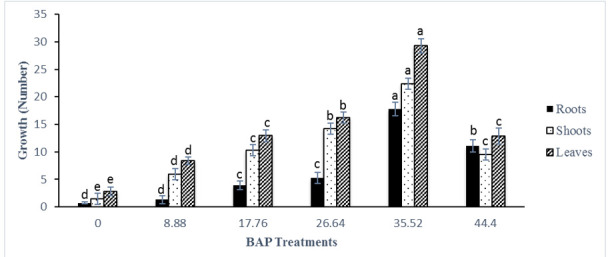
*K. parviflora* growth under different 6-benzyladenine (BAP) concentrations estimated by number of roots,shoots, and leaves after 9 weeks. Results represent mean ± standard error of 15 replicates. Means with the same letter for each measured parameter (roots, shoots, leaves) are not significantly different from each other, according to Duncan’s multiple-range test (*P* ≤ 0.05). Error bars represent standard error.

**Figure 4:**
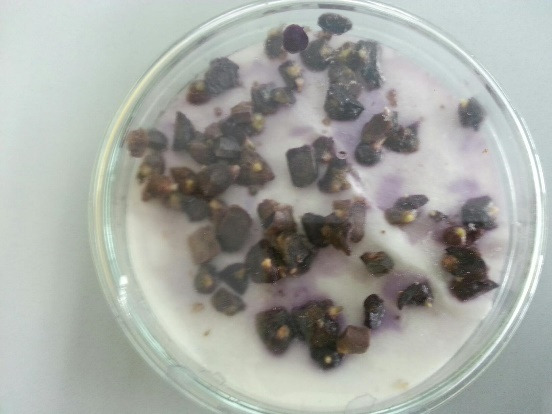

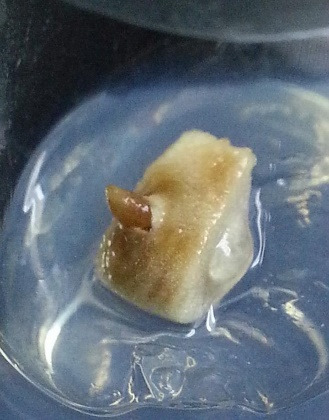

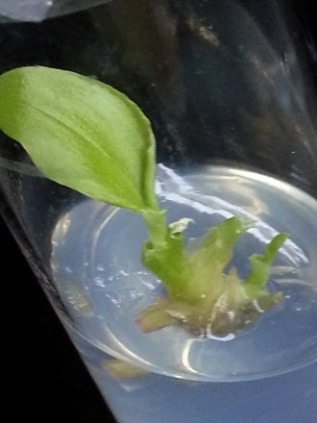

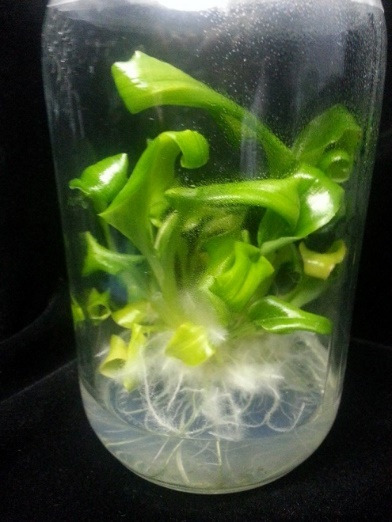

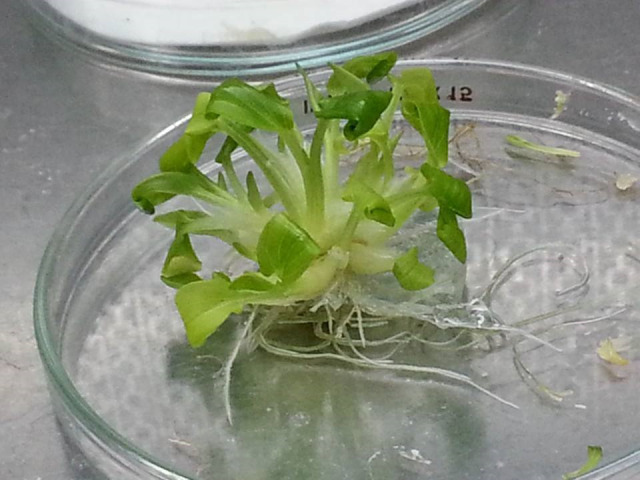

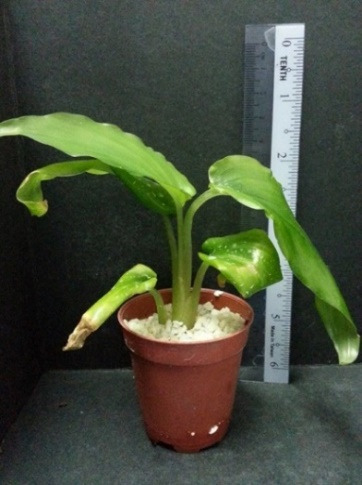
*K. parviflora* growth via *in vitro* propagation. (a) Excised explants; (b) Rhizome bud inoculated on MS media with treatments; (c) Shoot induction in rhizome bud under 35.52 μM BAP treatment; (d) Regeneration of shoots under 35.52 μM BAP treatment after 9 weeks of culture; (e) Root formation was spontaneous. Young plantlets were separated and planted in autoclaved perlite for acclimatisation. (f) Acclimatized plantlets after 2 weeks with several large leaves at 8cm in height. (Scale —— represents 1 cm).

Sterilisation of rhizome buds proved difficult with more than 76.4% of cultures affected with contamination prior to pre-sterilisation and use of mercury chloride in sterilisation procedure. Pre-sterilisation reduced contamination to lower than 15% and was adapted as an effective sterilisation technique (Table 1). Explants in the form of rhizome buds responded positively to all six BA treatments yet significant difference was observed among the treatments (Fig. 2). Explants showed continuous growth from 3 to 9 weeks of observation compared to control (only MS medium without any BA) explants that sprouted at very slow rates with none to minimal growth observed after 9 weeks of treatment (Fig. 2). BA with other five concentrations were successful for direct regeneration of *K. parviflora* from rhizome buds. The highest number of shoots was obtained for 35.52 μM BA after 9 weeks of treatment. A higher concentration of BA at 44.40 μM showed reduced growth from 3 to 9 weeks compared to BA treatments of 17.76, 26.64 and 35.52 μM (Fig. 3). Maximum shoot multiplication and proliferation was achieved with MS medium supplemented with 35.52 μM BA recording 22.4 ± 1.84 number of shoots per explant, 17.8 ± 1.72 number of roots per explant and 29.27 ± 1.30 number of leaves per explant (Fig. 3). Shoot multiplication was achieved in 9 weeks (Figs. 4b, c, d). MS medium with no BA (control) showed very low growth and minimal shoot multiplication. MS medium supplemented with 44.40 μM BA had high number of roots per explant (11.06 ± 2.12) but lower number of shoots (9.53 ± 2.13) and leaves (12.87 ± 2.41) per explant compared to 26.64 μM BA (Fig. 3). Rooting was spontaneous in all treatments. Shoots were separated (Fig. 4e) and acclimatised for 2 weeks with a 100% success rate. A 100% survival was achieved in acclimatisation using autoclaved perlite media under intermittent mist for 2 weeks. Acclimatised plantlets did not show any morphological abnormalities.

### Microrhizome Induction

**Figure 5:**
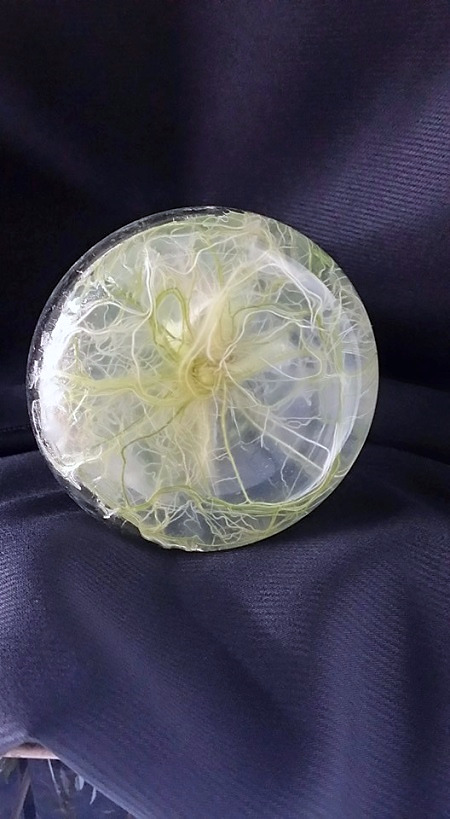

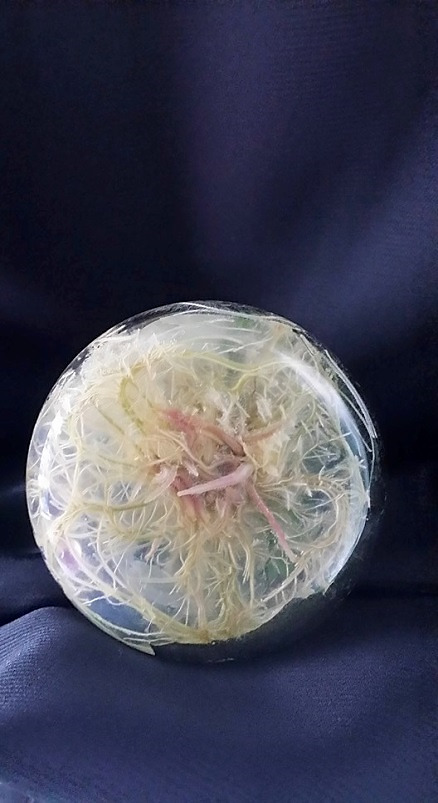

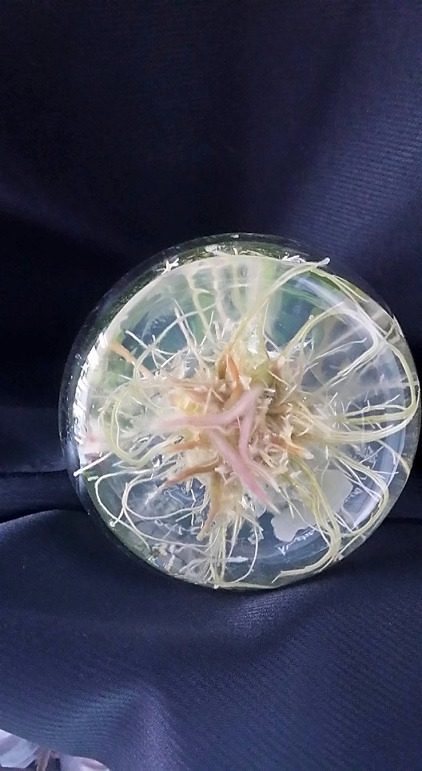
*K. parviflora* microrhizome induction: (a) 3% sucrose treatment did not form any red tuberous roots; (b) 6% sucrose treatment formed full root ball and red tuberous roots; (c) 9% sucrose treatment formed sparse roots and fewer red tuberous roots. (Scale —— represents 1 cm).

**Figure 6:**
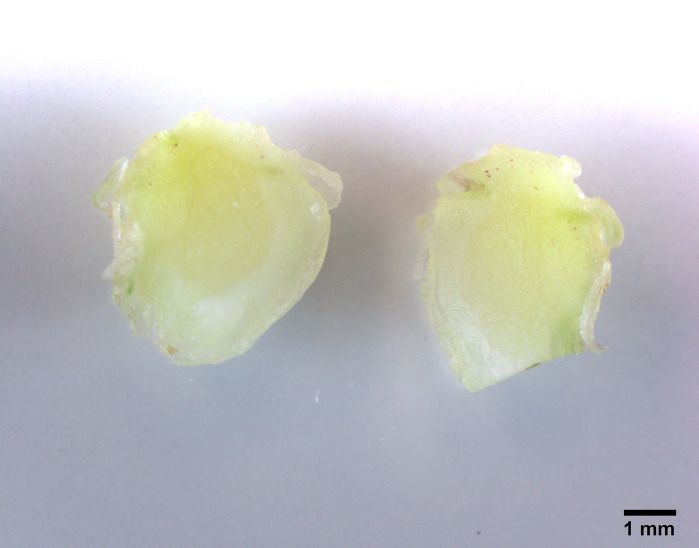

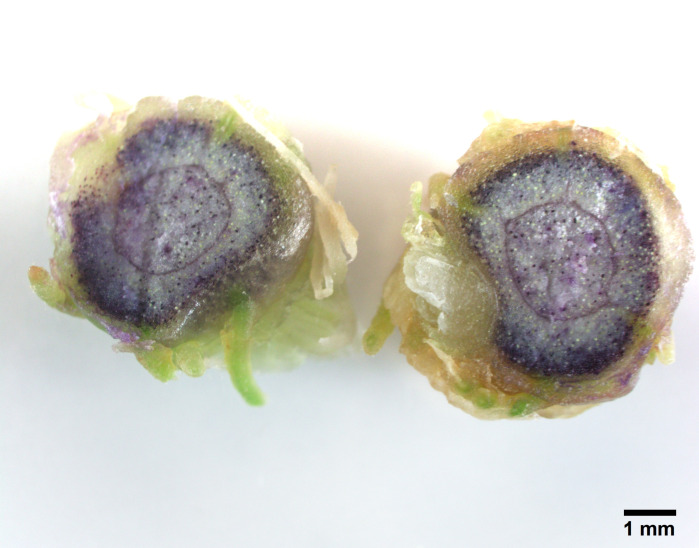

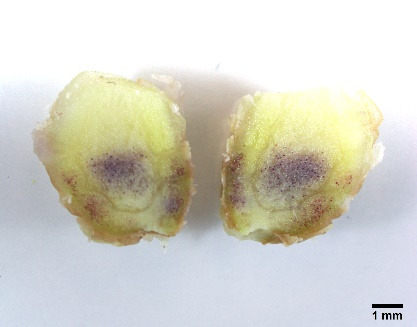
*K. parviflora* microrhizome cross section under 10x magnification (Olympus, Tokyo, Japan): (a) 3% sucrose treatment did not form any rhizome structure; (b) 6% sucrose treatment formed clear rhizome structure with mature colour; (c) 9% sucrose treatment formed rhizome structure without mature colour. (Scale —— represents 1 mm).

Young shoots regenerated from this experiment were separated and cultures in MS media with three different sucrose levels 3%, 6% and 9% for microrhizome formation. Based on root ball formation at the base of conical flasks (250 mL) with 50 mL media, 6% sucrose treatment showed compact roots filling most media and the appearance of thicker reddish tuberous roots (Fig. 5). In comparison 3% (w/v) sucrose treatment did not develop tuberous roots and 9% sucrose treatment did not grow as compact as 6% (w/v) (Fig. 5). This visual observation was further supported by quantitative data in Table 2. The 6% (w/v) sucrose treatment had higher number of shoots (8.5 ± 1.58) higher number of roots (25.1 ± 1.28) and highest number of microrhizomes (5.2 ± 0.78) (Table 2). Rhizome diameter (1.15 ± 0.24 cm) and weight per flask (35.7 ± 2.59 g) was also superior for this treatment. Microhizome formation was not observed for 3% (w/v) sucrose treatment (Table 2). A cross section of micrhizomes and shoot base showed well developed rhizome structure and colour for 6% sucrose treatment. Microrhizome in 9% (w/v) sucrose did not show mature colouring and in 3% sucrose did not show rhizome structure (Fig. 6). Acclimatisation was not required for plantlets with microrhizomes as they were directly transplanted in mist house with success.

## DISCUSSION

Establishment of aseptic culture from underground explant source is usually difficult due to higher contamination rates (Balachandran *et al.* 1990). Pre-sterilisation technique and sterilisation using mercury chloride significantly reduced contamination in this study (Table 1). In support of our observation, mercuric chloride (HgCl_2_) was also reported to be beneficial in reducing contamination in *in vitro* culture of other *Kaempferia* species (Shirin *et al.* 2000; Chirangini *et al.* 2005; Parida *et al.* 2010). Mahmoud and Al-Ani (2016) found that HgCl_2_ mainly has bactericidal action and is a more effective sterilant with better decontamination percentages compared to sodium hypochlorite.

In this study, treatment with HgCl_2_ was necessary and very effective for controlling contamination in *K. parviflora*. In the present study, BA treatment of 35.52 μM was optimum for shoot induction and regeneration with roots forming simultaneously within 9 weeks of culture. Similar results using BA were found in four out of five reports on *K. parviflora in vitro* propagation (Table 3). Two reports using rhizome buds as explants and MS media showed that BA was suitable for direct regeneration of *K. parviflora* (Mongkolchaipak *et al.* 2006; Prathanturarug *et al.* 2007). Mongkolchaipak *et al.* (2006) reported 31.08 μM BA treatment produced up to 4.5 shoots per explant in 8 weeks while rooting was increased using 5.37 μM α-napthalene acetic acid (NAA).

Prathanturarug *et al.* (2007) employed a slightly different method using a two-step direct regeneration method by culturing with BA treatment on regeneration media for 8 weeks and transferred to development media void of any plant growth regulators for 4 weeks. The 35.35 μM BA treatment showed best results of 7.16 ± 0.72 shoots per explant after 12 weeks of culture. It was also reported that addition of 2.69 μM NAA significantly reduced shoot formation. After 24 weeks and 2 cycles of culture 47.3 ± 3.4 shoots were obtained per explant for 35.35 μM BA treatment. Their research showed that optimum exposure to BA was effective in triggering shoot multiplication in *K. parviflora* but acclimatisation was not reported unlike our study. Shoot tips were also used as explant source previously (Dheeranupattana *et al.* 2003; Alveno 2012). In one of the earlier reports on *K. parviflora in vitro* culture, MS media supplemented with 2.68 μM NAA and 13.32 μM BA was successful in direct regeneration resulting in 2.4 shoots per explant after 4 weeks of culture (Dheeranupattana *et al.* 2003). More recently, Alveno (2012) reported short term rapid propagation of *K. parviflora* from young shoots in MS and ½MS media supplemented with BA treatments. The results were not significant among BA treatments and the highest number of shoots 1.77 shoots per explant resulted from 16.65 BA treatment in full MS media after 3 to 4 weeks of culture. These reports indicate that BA is an ideal PGR for direct regeneration of *K. parviflora*.

The results obtained from this study is comparative to previous research however more shoots per explant (22.4 shoots) was obtained using 35.52 μM BA under shorter culturing periods (9 weeks) indicating a preferable micropropagation method for quick production of *K. parviflora* plants. Interestingly, in our study it was observed that higher concentration BA showed reduced shoot number/length. This reduction of shoot growth with the higher concentrations of cytokinin after a certain extent corroborating the earlier observations of Shirin *et al.* (2000) in *K. galangal*. Rooting was spontaneous in our study and this was in concordance to observations of Prathanturarug *et al.* (2007) and Dheeranupattana *et al.* (2003). Dheeranupattana *et al.* (2003) also reported that acclimatisation was 100% successful after 2 weeks in 1:1:1 (v/v/v) of autoclaved sand, charred rice hull and coir dust media while Mongkolchaipak *et al.* (2006) reported that acclimatisation was achieved using soil for 4 weeks with a 98% success rate.

Our observation showed high success of 100% during acclimatisation of *in vitro* cultured *K. parviflora* plantlets on autoclaved perlite media. Rhizome yield is of economic importance for most Zingiberaceae. *In vitro* microrhizome induction has many benefits including disease free planting materials, easy transportation, easy transfer to field without acclimatisation, as living germplasm materials (Islam *et al.* 2004). There are many reports on microrhizome induction in Zingiberaceae family especially in medicinally important genus such as *Zingiber* (Sharma & Singh 1995; Chirangini & Sharma 2005; Tyagi *et al.* 2006; Zheng *et al.* 2008), and *Curcuma* (Nayak 2000; Shirgurkar *et al.* 2001, Islam *et al.* 2004, Lo-apirukkul *et al.* 2012) but limited in *Kaempferia* (Chirangini *et al.* 2005; Chithra *et al.* 2005). Sucrose has been shown to promote *in vitro* formation of storage organs such as tuber, bulbs, corms and rhizomes (Abbott & Belcher 1986; Hoque *et al.* 1996; Arora *et al.* 1996; Nayak 2000; Kim *et al.* 2003, Shirgurkar *et al.* 2001).

The results from the present investigation indicates that 6% sucrose is ideal for microrhizome induction. Bhat *et al.* (1994) reported that sucrose alone (9%–12%) was found to significantly influence rhizome formation in ginger (*Zingiber officinale*) compared to other among factors. Sucrose provides carbon energy that enhances organ formation especially storage organs that mostly store carbohydrates (Nayak 2000). In other Zingiberaceae species it was also found that sucrose in the range of 3%–9% was optimum for microrhizome induction (Nayak 2000; Shirgurkar *et al.* 2001; Rout *et al.* 2001; Chirangini & Sharma 2005; Chithra *et al.* 2005). Nayak and Naik (2006) observed that 6% sucrose successfully induced rhizome formation in turmeric (*Curcuma longa*) but a further increase of 9% decreased the percentage response in rhizome formation. It has also been reported in *Zingiber officinale* that after 3 months of culture supplemented with 10% sucrose plantlets exhibited symptoms of vitrification and bud decay (Archana *et al.* 2013). There are two known reports for microrhizome induction of *K. parviflora,* one using liquid culture system and another using high sucrose concentrations (Zuraida *et al.* 2014; Kafindra *et al.* 2015). Zuraida *et al.* (2014) reported that liquid MS medium supplemented with 4.44 μM BA and 1 NAA and 6% sucrose was most effective for microrhizome formation with 25.6 ± 3.4 g/flask produced in 3 months of culture. It was also reported that higher sucrose concentration to 9%–12% retarded plantlets and caused plantlet death. Kafindra *et al.* (2015) reported that MS medium with 9% sucrose void of BA treatment was ideal for microrhizome induction forming 5 rhizomes per plantlet after 8 weeks of culture. In comparison, our investigation found that semisolid MS medium supplemented with 6% sucrose formed 5.2 ± 0.78 rhizomes per explant with average weight of 35.7 ± 2.59 g/per flask after 9 weeks of culture. This method manipulating only one factor, sucrose was found to be economical and quick for producing large number of microrhizomes in a short time.

## CONCLUSION

The benefits of *in vitro* culture and microrhizome induction of medicinal plants motivated our investigation for *K. parviflora*. Traditional propagation of the plant to meet market demand proved challenging with several drawbacks including long dormancy periods, slow growth and the competitive use of rhizomes as yield and as planting seeds. *In vitro* direct regeneration of *K. parviflora* was successful using only BA in MS media almost within two months. The plantlets were then induced to form microrhizomes by manipulating sucrose concentrations. There are several reports on *in vitro* culture of *K. parviflora*, however our investigation highlights the manipulation of single factors in short time frame to produce a simple, economical and efficient dual-propagation method (in term of complete plantlets and microrhizome) for *K. parviflora*.

## Figures and Tables

**Table 1:** Sterilisation techniques for *Kaempferia parviflora* explants and contamination percentages.

Sterilisation	Components	Contamination percentage (%)
Technique 1	Without pre-sterilisation, Ethanol 70%, Clorox 10%, Clorox 20%, Distilled water	76.4
Technique 2	Pre-sterilisation, Ethanol 70%, Clorox 10%, Clorox 20%, Mercury chloride (HgCl_2_) 1%, Distilled water	12.0

*Note*: The second technique employed pre-sterilisation and mercury chloride was successful in reducing contamination to only 12%.

**Table 2:** Effect of sucrose on microrhizome induction in *K. parviflora.*

Sucrose (%)	Number of shoots per explant	Number of roots per explant	Number of microrhizomes per explant	Average diameter of microrhizomes (cm)	Fresh weight of microrhizomes (g/flask)
3	6.60 ± 1.07 ^b^	12.80 ± 2.48 ^c^	0 ^c^	0 ^c^	0 ^c^
6	8.50 ± 1.58 ^a^	25.10 ± 1.28 ^a^	5.20 ± 0.78 ^a^	1.15 ± 0.24 ^a^	35.70 ± 2.59 ^a^
9	6.00 ± 0.94 ^b^	19.10 ± 1.52 ^b^	3.40 ± 0.84 ^b^	0.56 ± 0.14 ^b^	11.30 ± 2.16 ^b^

*Note*: Results represent mean ± standard error of 10 replicates after 9 weeks of culture. Means with different letters in a column are significantly different from each other, per Duncan’s multiple-range test (*P* ≤ 0.05). Treatment of 6% sucrose was significantly superior to microrhizome production compared to 3% and 9% sucrose treatment.

**Table 3:** Summary of previous *In vitro* reports on *Kaempferia parviflora.*

In vitro research	Explant	Basal medium	Method	Duration (weeks)	Shoot multiplication		Rooting in vitro		Acclimatisation	
		
					PGRs (μM)	Result (shoots/explant)	PGRs (μM)	Result	Survival %	Duration
Dheeranupattana *et al.* (2003)	Shoot tips	MS	DR	4	2.68 NAA, 13.32 BA	2.4	n/a	Spontaneous	100%	2 weeks
Mongkolchaipak *et al.* (2006)	Rhizome buds	MS	DR	4–8	31.08 BA	4.2 to 4.5	5.37 NAA	Increased	98%	4 weeks
Prathanturarug *et al.* (2007)	Rhizome buds	MS	2 Step DR	16	35.52 BA or 4.54 TDZ	8.8 ± 0.6 or 12.8 ± 0.8	0 or 2.69 NAA	Spontaneous (0 NAA)	n.a.	n.a.
Alveno (2012)	Young shoots	MS, ½ MS	DR	3–4	16.65 BA	1.77	n.a.	n.a.	n.a.	n.a.
Zuraida *et al.* (2014)	Rhizome buds	MS	Callus Induction	13–14	4.55 2,4-D, 1.07 NAA	20% callus formation	n.a.	n.a.	n.a.	n.a.

*Note*: MS: Murashige and Skoog basal medium; DR: Direct regeneration method; BA: N6-benzyladenine; NAA: Naphthaleneacetic acid; TDZ: Thidiazuron; n/a: Non applicable
